# Importance of Diagnostic Imaging Training for Intensivists: Lessons Learned From a Case

**DOI:** 10.7759/cureus.55779

**Published:** 2024-03-08

**Authors:** Masayuki Akatsuka, Akira Hasebe, Naoya Yama

**Affiliations:** 1 Department of Intensive Care Medicine, Sapporo Medical University School of Medicine, Sapporo, JPN; 2 Center for Graduate Medical Education, Sapporo Medical University Hospital, Sapporo, JPN; 3 Department of Diagnostic Radiology, Sapporo Medical University School of Medicine, Sapporo, JPN

**Keywords:** physician, training, diagnostic imaging, intensive care medicine, education

## Abstract

Intensive care physicians are tasked with managing patients with complex organ disorders, necessitating a broad skill set and ongoing education. While simulation training often focuses on equipment handling, this case report highlights a critical instance of acute aortic dissection initially missed on imaging during intensive care unit (ICU) admission. An 86-year-old woman with multiple comorbidities presented with respiratory symptoms and electrolyte imbalances, ultimately requiring ICU admission. Despite initial inconclusive imaging and treatment for suspected conditions, further evaluation revealed a dissecting aneurysm of the descending aorta. This case underscores the importance of thorough diagnostic evaluation and ongoing vigilance, especially in older adults with multiple health conditions. It emphasizes the need for comprehensive education, including proactive training in image diagnosis, to recognize diverse medical presentations and potential complications. This case serves as a reminder of the evolving challenges in critical care and the necessity for continuous education and adaptability to ensure optimal patient outcomes.

## Introduction

Intensive care physicians often have to manage patients with serious organ disorders and therefore require a diverse range of knowledge and skills. The unique nature of every patient’s clinical course requires healthcare providers to be sufficiently trained through simulation education and hands-on seminars [1,2]. However, at present, much of this training is focused on handling equipment such as ventilators [3], extracorporeal membrane oxygenation machines [4], and echocardiography [5]. This article reports a case of acute aortic dissection that could not be detected on image diagnosis during intensive care unit (ICU) admission.

## Case presentation

An 86-year-old woman with a history of hypertension, nephrotic syndrome, and aortic valve stenosis initially sought medical attention for right knee pain and underwent temporary hospitalization and arthroscopic debris cleaning due to the presence of methicillin-sensitive *Staphylococcus aureus* in the synovial fluid. Post-surgery, she showed electrolyte imbalances, hyponatremia, and pleural effusion. She received steroid cover for suspected secondary adrenocortical insufficiency. However, the persistent anemia and respiratory symptoms ultimately necessitated admission to the ICU.

Clinical findings on ICU admission were as follows: blood pressure, 169/67 mmHg; heart rate, 100 beats/minute; respiratory rate, 24 breaths/minute; oxygen saturation, 99% with a high-flow nasal cannula (fraction of inspired oxygen, 0.7, 50 L/minute); partial arterial oxygen pressure, 150 mmHg; partial arterial pressure of carbon dioxide, 44 mmHg; pH, 7.54; base excess, 14 mmol/L; lactate, 12 mg/dL; sodium, 120 mEq/L; potassium, 2.3 mEq/L; and chlorine, 78 mEq/L (Table 1).

**Table 1:** Clinical findings on intensive care unit admission.

Variables	Measurements	Reference range
Blood pressure	169/67 mmHg	
Heart rate	100 beats/minute	
Respiratory rate	24 beats/minute	
Oxygen high-flow nasal cannula	Gas flow: 50 L/minute; fraction of inspired oxygen: 0.7	
Arterial blood gas analysis		
Partial pressure of oxygen	150 mmHg	80–100
Partial pressure of carbon dioxide	44 mmHg	35–45
pH	7.54	7.350–7.450
Base excess	14 mmol/L	-2.0–2.0
Lactate	12 mg/dL	5–14
Sodium	120 mEq/L	136–146
Potassium	2.3 mEq/L	3.4–4.5
Chlorine	78 mEq/L	98–106

Chest computed tomography (CT) revealed bilateral ground-glass opacities with reticular opacities in both lungs and bilateral pleural effusions. Treatment for acute respiratory failure, hyponatremia, hypokalemia, and adrenal insufficiency associated with exacerbation of acute heart failure was initiated. Gram-positive cocci were detected in the blood culture taken upon ICU admission.

**Figure 1:**
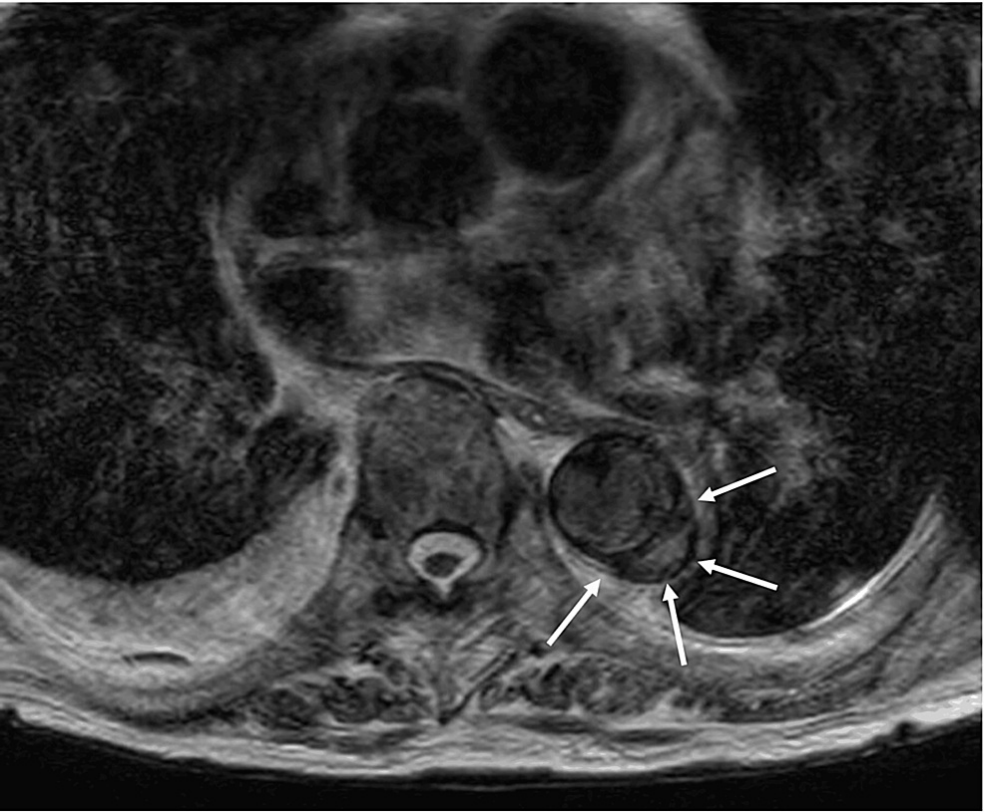
Magnetic resonance imaging of the descending aorta. T2 axial magnetic resonance imaging with white arrows showing a localized descending aortic dissecting aneurysm.

**Figure 2:**
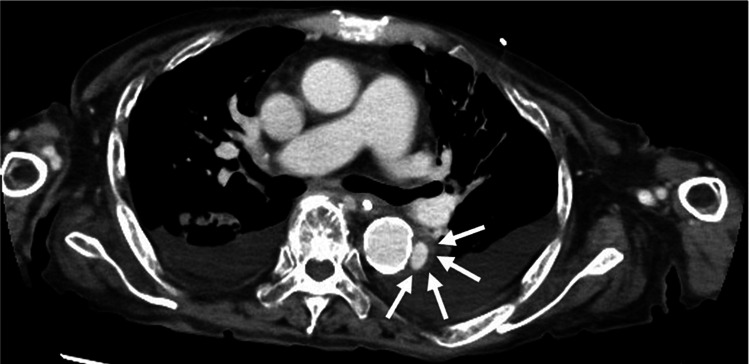
Contrast-enhanced computed tomography of the chest. The white arrows show a localized descending aortic dissecting aneurysm.

Contrast-enhanced CT to identify infection foci showed no obvious lesions. In the differential diagnosis of bacteremia, infective endocarditis and pyogenic spondylitis were considered. Echocardiography revealed no obvious lesions, and magnetic resonance imaging (MRI) indicated a diagnosis of pyogenic spondylitis. No suspicious findings were observed. Administration of diuretics and thoracentesis tended to improve the heart and respiratory failure, and the patient was discharged from the ICU on the sixth hospital day because her respiratory condition was stable with nasal cannula therapy at 3 L/minute and hemodynamics were stable. However, MRI interpretation by the radiology department indicated a dissecting aneurysm of the descending aorta (Figure 1). At this point, we looked back at the CT scans we had taken previously and found a similar dissecting aneurysm in the descending aorta (Figure 2). Consultation with a cardiovascular surgeon indicated that instead of a typical aortic dissection in the descending aorta, this was a tear in a heavily calcified part of the intima causing leakage of blood. Although the tear was limited, blood pressure management was required to avert a rupture.

## Discussion

In this case, two discernible findings have emerged, each bearing substantial implications for the realm of intensive care medicine. First, our report underscores the imperative integration of diagnostic imaging into the pedagogical framework designed for the enlightenment of intensive care practitioners. Second, the exigencies encountered in managing the aforementioned case highlight the escalating demand for an expanded repertoire of skills and knowledge within the purview of intensive care medicine.

This case accentuates a critical lacuna in the current training paradigm, suggesting a pressing need for augmented emphasis on the interpretation and application of diagnostic imaging techniques among intensivists. This finding underscores the necessity for intensivists to possess adept skills in interpreting imaging results to facilitate timely and accurate diagnosis. The significance of integrating imaging education into intensive care training programs has been emphasized [6]. Incorporating simulation-based training in radiology interpretation for critical care fellows leads to improved diagnostic accuracy and confidence levels among participants [7].

Moreover, the multifaceted clinical trajectory, characterized by a confluence of organ dysfunctions and diagnostic conundrums, underscores the indispensable requirement for intensivists to navigate an increasingly complex landscape with expertise. This case further delineates the demand for intensified skill sets and knowledge among intensive care physicians. Intensive care medicine has evolved into a dynamic discipline characterized by the complexity of patient presentations and the advent of advanced technologies and treatment modalities [8,9]. The evolving landscape necessitates a comprehensive understanding of diverse medical domains, ranging from cardiovascular physiology to infectious disease management. Moreover, the acquisition of advanced skills beyond traditional intensive care realms is needed to underscore the correlation between intensified training in critical care and improved patient outcomes [10]. The dynamic and multifaceted nature of critical care practice necessitates a holistic understanding of diverse medical domains to effectively address the evolving needs of critically ill patients.

## Conclusions

This case provides a reminder of the unique challenges encountered in each case and the need for a comprehensive and adaptable approach for optimal patient care. These lessons can contribute to the evolution of healthcare practices and underscore the importance of continuous education and adaptability to meet evolving medical challenges.
